# International perspectives on adherence and resistance to HIV antiretroviral therapy

**DOI:** 10.1186/1758-2652-13-S4-O2

**Published:** 2010-11-08

**Authors:** D Bangsberg

**Affiliations:** 1 Massachuesetts General Hospiral and Global Health Scholars, Massachusetts, USA

## 

Public health debates about providing HIV antiretroviral therapy to impoverished HIV+ populations are based on the relationship between adherence and risk of drug resistance to HIV antiretroviral therapy. Early justifications for withholding antiretroviral therapy from marginalized domestic populations, such as drug users and the homeless, were mistaken for two reasons. First, levels of adherence in marginalized populations were not much different than the general HIV+ population, and second, early single protease-based antiretroviral therapy lead to drug resistance predominately in highly (80-95%) adherent individuals. In retrospect, HIV antiretroviral drug resistance during the first decade of effective therapy was not driven by poor adherence, but rather by the fact that early regimens were not potent enough to fully suppress the virus in patients who took most, if not all, of their medications. The introduction of more potent ritonavir-boosted protease inhibitor and non-nucleoside reverse transcriptase inhibitor regimens have shifted this relationship towards full viral suppression and cessation of drug resistance at high levels of adherence.

Similar concerns slowed the provision of HIV antiretroviral therapy in resource-limited settings based on the expectations that extreme poverty would lead to poor adherence and the global spread drug resistant virus. Data from most studies indicate that these concerns were overstated. Individuals in resource-limited settings consistently take >90% of their medication, compared to 70% in resource-rich settings. Successful adherence can be explained on the practice of individuals leveraging their social capital to ask friends and family to overcome structural and economic barriers to treatment adherence. Furthermore, full viral suppression is possible in antiretroviral naïve patients on non-nucleoside reverse transcriptase inhibitors regimens, typical of resource-limited settings, at moderate levels of adherence. Rather, the risk of drug resistance in resource-limited settings appears to be more related interruptions in therapy due to structural and financial barriers to drug supply and distribution. These interruptions in therapy lead to nevirapine monotherapy as nucleoside antiretroviral drug levels (stavudine and lamivudine) decay more rapidly than non-nucleoside reverse transcriptase inhibitor drug levels (nevirapine) following a treatment interruption ("the nevirapine tail"). While efforts to sustain and prevent declines in adherence will be important in resource-limited settings, resistance will have less to do with forgotten doses, than ensuring a reliable drug supply and distribution system.

